# Preparation of Se-doped Co/Ni bimetallic composite carbon nanotubes and investigation of their oxidation properties

**DOI:** 10.1039/d5ra08405a

**Published:** 2026-02-24

**Authors:** Wenbin Jia, Pengju Wu, Chao Wu, Meng Yang, Ying Wu

**Affiliations:** a Tarim University China

## Abstract

Developing highly efficient and stable selenium-doped transition metal bimetallic catalysts to replace precious metal electrocatalysts for the alkaline oxygen evolution reaction (OER) remains an ongoing challenge. This paper reported the synthesis of a highly efficient redox-active selenium-doped bimetallic composite catalyst (Se–CoNi@NCNTs) *via* a one-step pyrolysis method using cobalt-nickel and g-C_3_N_4_ as precursors. The Se–CoNi@NCNTs catalyst delivered a current density of 10 mA cm^−2^ at a low overpotential of merely 289 mV. Furthermore, in a 1 M KOH solution, the Tafel slope for electron transfer kinetics was determined to be 66.94 mV dec^−1^. It exhibited excellent stability during 100 h of alkaline overcurrent operation, with negligible electrocatalytic current density loss during 100 h of continuous electrolysis. Enhanced electrochemical performance was observed in 1 M KOH + 0.33 M urea electrolyte, requiring only a low overpotential of 98 mV to deliver a current density of 10 mA cm^−2^. Utilizing *in situ* catalysis of g-C_3_N_4_ precursors with metallic nanoparticles to generate nitrogen-doped carbon nanotubes (NCNTs) endows the composite CoNiSe compound with high intrinsic activity and conductivity, enabling highly efficient water oxidation.

## Abstract 2

Developing highly efficient and stable selenium-doped transition metal bimetallic catalysts to replace precious metal electrocatalysts for the alkaline oxygen evolution reaction (OER) remains an ongoing challenge.

## Graphical abstract

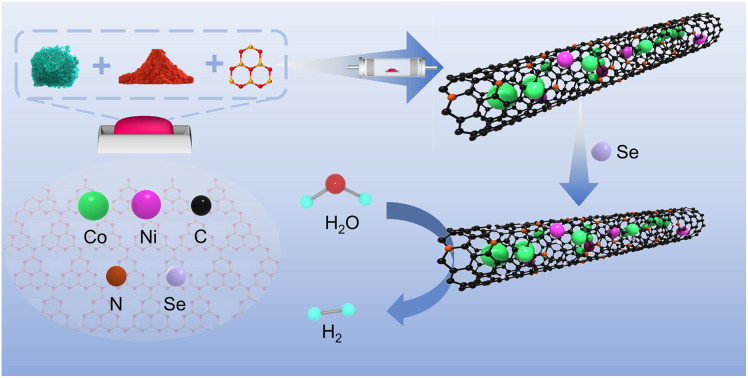

## Introduction

Hydrogen is widely regarded as an ideal energy carrier due to its zero carbon emission, recyclability, and high energy conversion efficiency.^1^ Electrochemical water splitting represents a direct steam hydrogen production technology, offering advantages such as environmental friendliness, high product yield, and high purity.^2^ Electrochemical water splitting comprises two half-reactions: the hydrogen evolution reaction (HER) and the oxygen evolution reaction (OER).^3,4^ The OER represents the pivotal half-reaction in water electrolysis and is regarded as a critical process for numerous promising renewable energy conversion and storage systems, including metal–air batteries and renewable fuel cells. The OER constitutes a bottleneck process characterized by slow four-proton coupled electron transfer kinetics and substantial activation energy barriers, thereby limiting overall hydrogen production efficiency.^5–7^ Currently, established electrocatalysts for OER are Pt-based and Ir-based materials.^8,9^ However, the high cost and inherent scarcity of precious metals severely constrain the future widespread application of these electrocatalysts. Consequently, developing highly active, stable, and low-cost electrocatalysts to replace precious metal-based materials represents a crucial step towards the future hydrogen economy.^10,11^ In recent years, bifunctional electrocatalysts based on transition metals (Mn, Fe, Co, and Ni) and their derivatives (carbides, oxides, sulfides, phosphides, hydroxides, and mixed-metal alloys) have demonstrated considerable potential.^12–18^ Under specific conditions, some exhibit electrocatalytic performance comparable to or even surpassing that of Pt/C for OER.^19^ However, their low catalytic activity, poor electronic conductivity, and solubility in aqueous electrolytes severely limit the efficiency and stability of water electrolysis.

Recently, carbon nanotube (CNT) materials have garnered extensive attention due to their regular tubular structure and excellent conductivity, finding widespread application in lithium-ion batteries, supercapacitors, and electrocatalysis.^20–22^ Given their large surface area, carbon nanotubes possess favourable surface chemistry and metal–N–C structures, making them promising candidates for integration with transition metal oxides to form highly efficient electrocatalytic electrodes.^23,24^ Wang *et al.* reported mesoporous thin-walled CuCo_2_O_4_@CNTs as a bifunctional oxygen electrocatalyst for rechargeable ZABs.^25^ Xia *et al.* reported NiFe_2_O_4_ nanoparticles anchored on CNTs as bifunctional electrode materials for hydrogen (H_2_) and oxygen (O_2_) evolution reactions.^26^ As is well known, conventional carbon nanotube synthesis methods, such as arc discharge, laser ablation, and chemical vapour deposition, invariably face challenges including low yields, costly synthesis equipment, and high expenses for large-scale preparation and application. The incorporation of transition metals within CNTs also poses a significant challenge in fabricating CNT nanostructures, with transition metals being confined by encapsulating metal ions within the nanotubes. Cobalt (Co) and nickel (Ni) nanoparticles can catalyse the growth of carbon nanotubes. Moreover, the corresponding Ni–N–C and Co–N–C sites within nitrogen-doped CNTs are recognised as active sites for O_2_ activation.^27–30^ Furthermore, Co- and Ni-based sites represent promising OER candidates, as they promote conversion to highly active phases such as CoOOH or NiOOH. This paper therefore proposes a solid-state synthesis strategy to directly convert solid graphitic carbon nitride (g-C_3_N_4_) into CNTs containing metallic particles. Moreover, further doping of nitrogen into carbon materials (NC) can effectively enhance OER activity, as the doped nitrogen attracts electrons from carbon atoms, and the induced changes in electronic structure can enhance O_2_ adsorption. Such NC materials are predominantly designed through the pyrolysis of nitrogen-rich carbon sources at elevated temperatures. Indeed, by mixing metal precursors with carbon sources, the strong interaction between metals and nitrogen atoms leads to the formation of M–NC structures (where M denotes the metal) upon annealing, which can be employed to retain nitrogen within the carbon framework.^31^ Moreover, the metal within M–NC can modulate the electronic structures of both nitrogen and carbon to facilitate strong interactions with O_2_, significantly enhancing the OER. Furthermore, high conductivity in materials such as carbon and metal is crucial for maximising electrocatalytic performance by enabling rapid electron transfer to or from the active sites. The choice of g-C_3_N_4_ as a precursor, rather than commercial CNTs, is deliberate and integral to the catalyst design. g-C_3_N_4_ provides a reactive source of both carbon and nitrogen, enabling the *in situ* formation of nitrogen-doped CNTs (NCNTs) during pyrolysis. This process simultaneously facilitates the creation of strong Co/Ni–N–C bonds and the uniform encapsulation of metal nanoparticles within the CNT walls. This integrated structure ensures strong metal–support interaction, enhanced conductivity, and high stability, which are challenging to achieve through conventional blending with pre-synthesized CNTs.

Transition metal selenides (TMS) have emerged as a novel family of electrocatalysts, offering alternatives to current precious metals or serving as replacement candidates due to their tunable bandgaps, atomic environments, electronic structures, and multiphase architectures with distinct conductivities.^32^ Specifically: modifications to TMS synthesis conditions or electronic structures readily modulate corresponding intrinsic catalytic activity.^11,33,34^ Catalytic activity correlates closely with overall phase structure, with semiconductor and metallic phases typically yielding distinct catalytic behaviours.^35^ Conversely, selenium's vacant 3d orbital energy level lies closer to its 3s and 3p orbitals, facilitating easier bonding with transition metal atoms compared to sulphur and oxygen. Consequently, TMS compounds generally exhibit heightened metallic character, which enhances electron transport and electrocatalytic reactions.^36^ To further elevate the material's oxygen evolution reaction (OER) performance, selenization treatment was applied to the material based on a cobalt–nickel bimetallic composite.

**Fig. 1:**
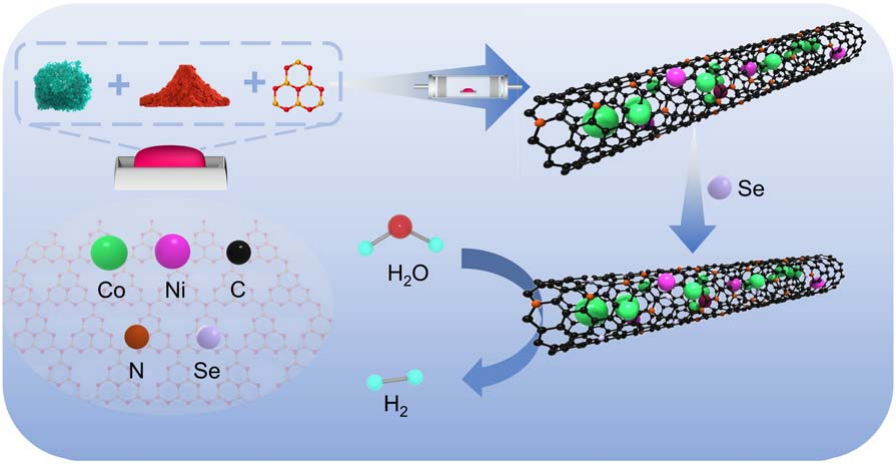
Schematic diagram of the synthesis of Se–CoNi@NCNTs composite material.

In this work, CoNi@NCNTs were synthesised *via* a high-temperature one-step pyrolysis method to achieve an efficient, stable, and low-potential-drive oxidation electrocatalyst. The prepared material precursor was annealed in an argon atmosphere to cross-link and consolidate the structure. During annealing, some Co and Ni elements aggregated at elevated temperatures to form CoNiNPs, which catalysed g-C_3_N_4_*in situ* to generate nitrogen-doped carbon nanotubes. The remaining Co and Ni existed within the nanotubes as Co–N and Ni–N complexes. Conversely, Se doping favours the formation of unique active sites that enhance catalytic performance, exhibiting particularly pronounced effects in metal-based catalysts incorporating Se as a functional heteroatom. Consequently, Se was introduced into the CoNi@NCNTs framework to modulate the adsorption of H and OH intermediates and reduce the energy barrier for the Volmer reaction. Although previous studies have reported the application of transition metal selenide/carbon nanotube composites in the OER, this work employs a one-step pyrolysis combined with vapor-phase selenization strategy to achieve uniform encapsulation and structural coupling of Se–CoNi nanoparticles within nitrogen-doped carbon nanotubes, significantly enhancing the material's conductivity, accessibility of active sites, and long-term stability. Furthermore, this study is the first to apply this material to a urea-assisted water electrolysis system, enabling highly efficient UOR and OER at low potentials, thereby offering new insights for the resource utilization of urea-containing wastewater (Fig. 1).

## Experimental section

### Materials

Cobalt(ii) nitrate hexahydrate (Co(NO_3_)_2_·6H_2_O) and nickel(ii) nitrate hexahydrate (Ni(NO_3_)_2_·6H_2_O) were both purchased from Kalmar Reagent Co., Ltd. Melamine (C_3_H_6_N_6_) was purchased from Aladdin Reagent Co., Ltd. Urea (CO(NH_2_)_2_) was purchased from Tianjin Fengchuan Chemical Reagent Technology Co., Ltd. Selenium powder (Se) was purchased from Shanghai Shanpu Chemical Co., Ltd. Anhydrous ethanol was purchased from Tianjin Zhiyuan Chemical Reagent Co., Ltd. Nafion solution (5 wt%) was purchased from Beijing Jingke Keyi Scientific Instrument Co., Ltd. All chemicals were of analytical grade and were not further purified prior to use.

### Synthesis

#### Synthesis of g-C_3_N_4_

Weigh a specified quantity of melamine into a ceramic crucible. Place the crucible in a muffle furnace and set the programme to heat at 5 °C min^−1^ to 550 °C. Maintain this temperature for 3 hours. After natural cooling, remove the crucible to obtain g-C_3_N_4_. Grind the obtained g-C_3_N_4_ thoroughly, pass through a 200-mesh sieve, and collect the product for subsequent use.

#### Synthesis of X@NCNTs (X = Co, CoNi)

X@NCNTs were prepared *via* a high-temperature one-step pyrolysis method. Weigh 1 g of g-C_3_N_4_ and 0.882 g of Co(NO_3_)_2_·6H_2_O into a beaker. Add 5 mL of anhydrous ethanol to the beaker, stir thoroughly, then sonicate for 10 minutes. Transfer the sonicated mixture to an agate mortar and grind until the material is dry and uniformly mixed. The resulting powder was loaded into a nitrogen-filled tube furnace. The furnace was programmed to heat at 5 °C min^−1^ to 700 °C (with holding times of 2 hours at 750 °C, 800 °C, 850 °C, and 900 °C). After cooling the tube furnace, shut off the nitrogen supply and remove the porcelain boat. Thoroughly grind the material and sieve through a 200-mesh screen to obtain Co@NCNTs. The preparation method for CoNi@NCNTs is identical to that for Co@NCNTs: weigh 1 g g-C_3_N_4_, 0.88 g Co(NO_3_)_2_·6H_2_O, and 0.89 g Ni(NO_3_)_2_·6H_2_O into a beaker, with subsequent steps the same. By adjusting the ratio of Co(NO_3_)_2_·6H_2_O to Ni(NO_3_)_2_·6H_2_O (3 : 0, 3 : 1, 2 : 1, 1 : 1), the resulting materials were designated as Co@NCNTs, CoNi@NCNTs-3 : 1, CoNi@NCNTs-2 : 1, and CoNi@NCNTs-1 : 1 respectively.

#### Synthesis of Se–CoNi@NCNTs

The selenide layer was deposited onto the surface of CoNi@NCNTs *via* a vapour-phase selenization method. One hundred milligrams of the prepared CoNi@NCNTs and 400 milligrams of selenium powder were placed in two separate porcelain boats within a tube furnace, with the boat containing selenium positioned upstream. The furnace was programmed to heat at 10 °C min^−1^ to 700 °C, followed by 1 h holding under a controlled nitrogen flow rate. Upon cooling to room temperature, the Se–CoNi@NCNTs nanocomposite material was obtained. Materials with adjusted selenium powder additions (100 mg, 300 mg, 500 mg) were designated Se–CoNi@NCNTs-1, Se–CoNi@NCNTs-3, and Se–CoNi@NCNTs-5 respectively.

### Characterization

The surface morphology of the catalyst material was observed using a Hitachi Regulus 8100 scanning electron microscope (SEM). The crystalline phase of the samples was investigated by X-ray diffraction (XRD) analysis using a BRUKER D8 Advance with CuKa radiation and a scanning range of 10° to 80°. The tube voltage was 45 kV, and the current was 4 mA. The particle size of the material was tested using Malvern Instruments Ltd instrument model MAL-1180433. X-ray photoelectron spectroscopy (XPS) modelled as Thermo Fisher Scientific K-Alpha was used to study the molecular structure and chemical state of the materials.

### Electrochemical measurements

Electrocatalytic testing of the urea oxidation reaction (UOR) and oxygen evolution reaction (OER) was conducted using a CHI 760E electrochemical workstation (Shanghai Chenhua Instruments Co.,Ltd) in a standard three-electrode configuration. The material was clamped onto a platinum sheet electrode holder to serve as the working electrode, while a platinum wire electrode and a Hg/HgO electrode were employed as the counter electrode and reference electrode respectively. All potentials were converted to reversible hydrogen electrode (RHE) scale using the following formula: *E*(RHE) = *E*(Hg/HgO) + 0.198 + 0.059 × pH. For UOR measurements, the electrolyte comprised 1.0 M KOH with or without 0.33 M urea. Prior to electrochemical testing, electrodes were activated *via* two consecutive cycles of 20 cyclic voltammetry (CV) scans at a scan rate of 100 mV s^(−1) to ensure stability. Linear sweep voltammetry (LSV) was conducted at a scan rate of 5 mV s^(−1) to evaluate electrocatalytic activity. Working electrode preparation: Pre-treated nickel foam (NF) was cut to dimensions of 1.5 cm × 1 cm (length × width). The NF surface organic impurities were first removed by ultrasonic treatment in acetone for 10 minutes. Subsequently, the NF surface oxidised impurities were treated by ultrasonication in 3 mol L^−1^ hydrochloric acid solution for 10 minutes. Finally, it was ultrasonicated with deionised water and ethanol separately for 10 min each, then dried for later use. Five milligrams of the catalyst was mixed with 2 µL binder and 100 mL anhydrous ethanol thoroughly, ultrasonicating for 30 min. The resulting slurry was applied onto the pretreated NF and dried for later use.

## Results and discussion

### Microstructural and structural characterisation

**Fig. 2:**
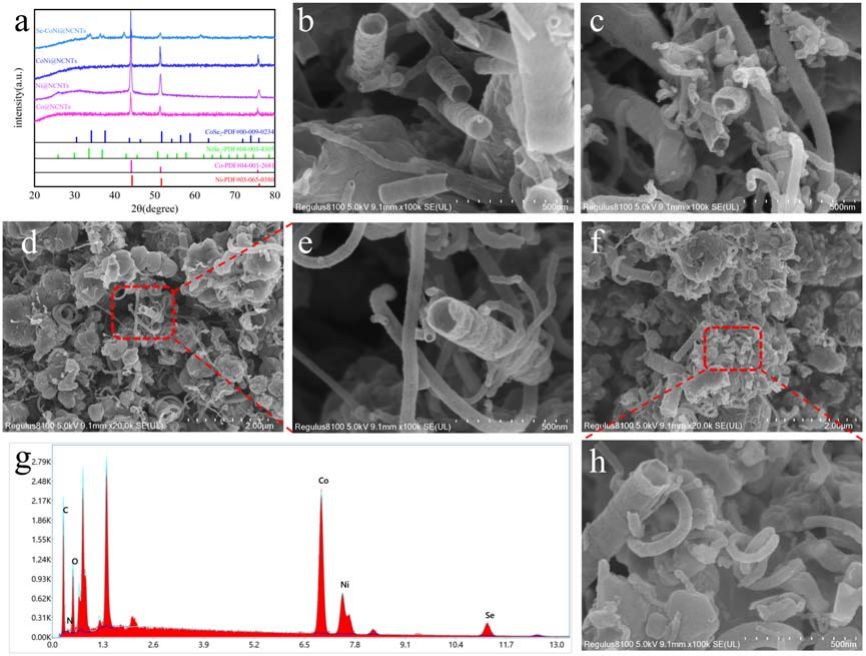
(a) XRD pattern of the material; (b–d) SEM images of Co@NCNTs, Ni@NCNTs, and CoNi@NCNTs; (e and f) SEM images of Se–CoNi@NCNTs; (g and h) EDS patterns of Se–CoNi@NCNTs.

The structure of the catalysts was analysed using X-ray diffraction (XRD). As shown in Fig. 2(a), the X-ray diffraction patterns of Co@NCNTs, Ni@NCNTs, and CoNi@NCNTs all exhibit three distinct peaks at approximately 44°, 51°, and 75°, matching well with the metal Co phase (PDF#04-001-2861) and Ni phase (PDF#03-065-0380). Generally, metallic phases of certain elements exhibit superior conductivity compared to their oxide counterparts. The presence of metallic Co and Ni nanoparticles indicates that a portion of the cobalt and nickel ion precursors were reduced during pyrolysis. Some cobalt and nickel nanoparticles were encapsulated within the carbon walls of the nanotubes. Furthermore, the X-ray diffraction peaks of Se–CoNi@NCNTs highly overlap with the NiSe_2_ and CoSe_2_ PDF standard patterns. Compared to the composite before selenium doping, the diffraction peak intensities are significantly reduced, indicating that selenium doping reacts with a portion of the metallic nanoparticles to form NiSe_2_ and CoSe_2_. The morphology of the catalyst was characterized using scanning electron microscopy (SEM). The SEM image in Fig. 2 reveals an abundant and uniform hollow carbon nanotube structure. The elongated hollow tubular architecture offers substantial advantages in expanding the active surface area, facilitating electron transfer, and reducing the distance between reactants and the active sites. Following annealing under a flowing N_2_ atmosphere, structurally well-defined CoNi carbon nanotubes were obtained. For single Ni and Co doping cases, the singly doped Ni@NCNTs and Co@NCNTs exhibited similar nanotube morphologies. Finally, following further Se doping in an N_2_ atmosphere, the resulting Se–CoNi@NCNTs retained the original array's outline, with some nanoparticles encapsulated within the nanotubes and exhibiting relatively rough surfaces. Furthermore, as demonstrated by EDS analysis, Co, Ni, and Se elements were found to be composite within the material in corresponding proportions. This also confirms that the Co and Ni metals were coated by layers of NCNTs, whilst the Se element was uniformly doped throughout. Previous studies indicate that zero-valent Ni and Co can promote and catalyse carbon nanotube formation. Both Ni^2+^ and Co^2+^ present in the material can be reduced to zero-valent states by carbon during calcination at 850 °C, forming Co and Ni metallic nanoparticles. To further demonstrate the crucial role of Ni^2+^ and Co^2+^ in carbon nanotube formation, the same synthesis process was conducted at varying Ni^2+^ and Co^2+^ ratios. CoNi@NCNTs synthesised at low Co^2+^ concentrations exhibited similar nanotube-like structures, yet the resulting nanotubes were comparatively shorter and incomplete, with inferior catalytic performance. To optimize the Co/Ni ratio, we synthesized CoNi@NCNTs with different Co : Ni ratios (3 : 0, 3 : 1, 2 : 1, 1 : 1) and systematically evaluated their OER performance. As shown in Fig. S1(b), the sample with a Co : Ni ratio of 3 : 1 exhibited the lowest overpotential (289 mV at 10 mA cm^−2^), indicating the strongest synergistic effect and optimal electronic structure at this ratio. This may be attributed to the fact that the electronic coupling between Co and Ni reaches an optimal balance under this ratio, which facilitates OH^−^ adsorption and O–O bond formation. At a Co : Ni ratio of 3 : 1, the resulting CoNi@NCNTs exhibited improved catalytic performance with relatively more complete samples. At 850 °C, g-C_3_N_4_ transforms into N-doped graphitic carbon. And demonstrates good OER performance, as shown in Fig. S1(a). The formed Co and Ni metallic nano particles promote and catalyse the conversion of carbon species in the N-doped graphite into nanotube structures, ultimately yielding CoNi@NCNTs with multiple heterointerfaces. Meanwhile, selenium doping creates unique active sites that enhance catalytic performance. The larger specific surface area and mesoporous structure likely provide more catalytic active sites and offer a favourable mass transfer pathway. We systematically investigated the influence of Se content on the material's performance (Fig. S1(c)). When the amount of selenium powder was 300 mg, the material exhibited the best OER activity (*η*_10_ = 344 mV), while either excessive or insufficient selenium resulted in performance degradation. This may be attributed to the fact that moderate Se doping can effectively regulate the electronic structure of the metals while avoiding the blockage of active sites by excessive inactive Se species.

**Fig. 3:**
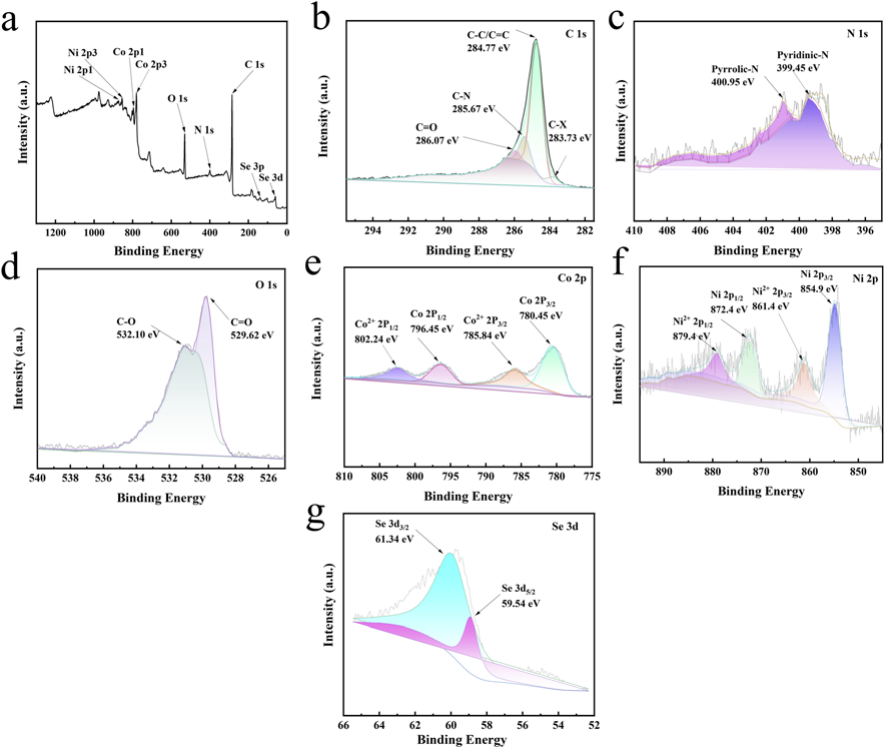
XPS spectra of Se–CoNi@NCNTs: (a) survey scan, (b) C 1s, (c) N 1s, (d) O 1s, (e) Co 2p, (f) Ni 2p, (g) Se 3d.

The structure of Se–CoNi@NCNTs was characterized using X-ray photoelectron spectroscopy. As shown in Fig. 3, XPS was employed to determine the elemental composition and chemical states of Se–CoNi@NCNTs. Fig. 3(a) illustrates the XPS spectrum of Se–CoNi@NCNTs, revealing the presence of six principal elements (C, N, O, Co, Ni, and Se). This confirms successful Se doping into the carbon nanotube structure, whilst the oxygen peak arises from oxidation of surface alloys in air exposure. The high-resolution C 1s spectrum (Fig. 3(b)) exhibits four peaks at 283.73 eV, 284.77 eV, 285.67 eV, and 286.07 eV, corresponding to C–X (Co or Ni), C–C/C–C, C–N, and C–O bonds respectively. The N 1s spectrum (Fig. 3(c)) exhibits two peaks at 399.45 eV and 400.95 eV, representing graphitic nitrogen and carbon–nitrogen bonds respectively, arising from N-doped carbon nanotubes and N-doped carbon hollow microspheres. Moreover, the high-resolution Co 2p spectrum exhibits a similar pattern (Fig. 3(e)), revealing two distinct Co states associated with Co (780.45 eV and 796.45 eV) and Co^2+^ (785.84 eV and 802.24 eV), corresponding respectively to Co 2p^1/2^ and Co^2+^ 2p^3/2^. As depicted in Fig. 3(f), the doublets at 861.4 eV and 879.4 eV are assigned to Ni^2+^ in the selenium structure, whilst the doublets at 852.91 eV and 869.98 eV are attributed to nickel oxide species formed by surface oxidation. Furthermore, the binding energies of Co 2p and Ni 2p in CoNi exhibit positive shifts compared to the pristine state, indicating modulated electronic structures of Co and Ni. These results demonstrate strong electronic coupling and interactions between Co and Ni metallic nano particles and CNTs, which favourably accelerate interfacial electron transfer and enhance OER activity. The detection peak at 530.9 eV confirms the presence of oxygen species on the catalyst surface, corresponding respectively to metal–oxygen bonds and surface-adsorbed hydroxyl groups. This indicates that the Se–CoNi@NCNTs surface is partially hydroxylated, consistent with the observed Ni–OOH. The abundance of surface adsorbed OH groups endow the catalyst with hydrophilicity, prolonged exposure to readily accessible active sites, and high ionic permeability. These properties are crucial for surface conductivity and catalyst intermediate (OH charge carrier) interface interactions during the water oxidation reaction. The peak detected at 531.8 eV is attributed to a surface-derived Se phase.

**Fig. 4:**
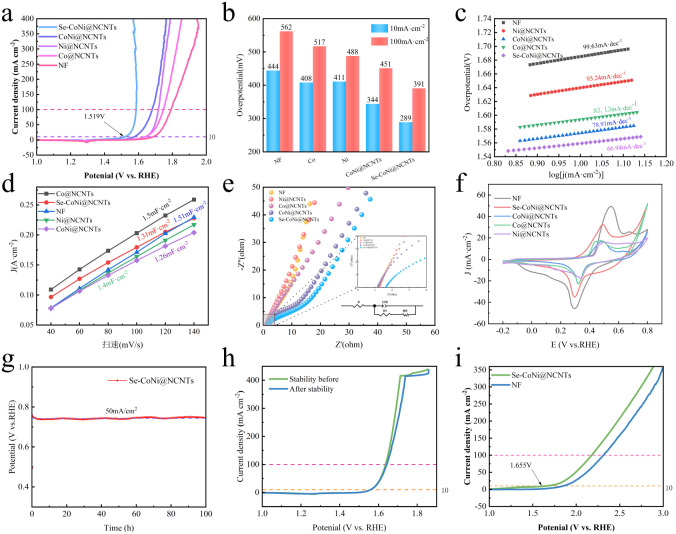
OER performance of NF, Co@NCNTs, Ni@NCNTs, CoNi@NCNTs, and Se–CoNi@NCNTs (a) LSV curves of the five materials, (b) overpotential performance comparison of the five materials, (c) Tafel slope plots of the five materials, (d) *C*_dl_ plots for the five materials, (e) EIS plots for the five materials, (f) CV plots for the five materials, (g) 100-hours stability test of Se–CoNi@NCNTs material, (h) LSV comparison before and after the 100-hours stability test of Se–CoNi@NCNTs material, (i) overall water splitting performance of Se–CoNi@NCNTs material.

The prepared Se–CoNi@NCNTs were subjected to electrocatalytic OER testing in a 1 M KOH solution at room temperature using a three-electrode configuration. Linear sweep voltammetry (LSV) was employed to evaluate OER overpotential activity. The effects of calcination temperature and varying CoNi ratios on the material were first investigated under a nitrogen atmosphere, as illustrated in Fig. S1. Results indicated that the optimum calcination temperature was 850 °C. As depicted in Fig. 4(a), Se–CoNi@NCNTs exhibited superior OER performance compared to other materials, achieving a low overpotential of merely 289 mV at 10 mA cm^−2^. Fig. 4(b) displays the overpotentials of different catalysts at specific current densities (10 mA cm^−2^ and 100 mA cm^−2^). Notably, the Se–CoNi@NCNTs material requires only a very low overpotential (359 mV) to generate a high current density of 100 mA cm^−2^. The overpotential results indicate that the Se–CoNi@NCNTs catalyst exhibits the highest catalytic activity. Compared to Co@NCNTs, Ni@NCNTs, and CoNi@NCNTs, Se–CoNi@NCNTs demonstrates a lower Tafel slope (66.9 mV dec^−1^), suggesting rapid surface kinetics (Fig. 4(c)). Based on the above data, the activity of the single-metal catalysts is significantly lower than that of the bimetallic Se–CoNi@NCNTs, underscoring the crucial role of the Co–Ni synergistic effect. Moreover, CoNi@NCNTs requires a higher overpotential than Se–CoNi@NCNTs, demonstrating that selenium doping electronically modulates the metal sites and enhances their intrinsic activity. The XPS and SEM analyses presented in Fig. S4 confirm that the catalyst surface reconstructs into Co/Ni (oxy)hydroxides during OER. Therefore, the active sites are likely the (oxy)hydroxide species generated *in situ* on the surface. The electrochemical surface area (ECSA) for OER is proportional to the electrochemical double-layer capacitance (*C*_dl_), as shown in Fig. S2. As shown in Fig. 4(d), the *C*_dl_ value of Se–CoNi@NCNTs was 1.31 mF cm^−2^, slightly lower than other materials. This indicates that Se–CoNi@NCNTs inherently possess a smaller electrochemical surface area, yet exhibit high intrinsic activity, delivering excellent catalytic performance despite the reduced *C*_dl_. Fig. 4(e) displays the corresponding Nyquist plots (EIS).

Furthermore, Se–CoNi@NCNTs demonstrate good electrochemical stability at 50 mA cm^−2^, as shown in Fig. 4(g) and (h). Fig. S4, the high-resolution XPS spectra of Co 2p and Ni 2p for the sample after the 100-hours stability test showed a significant positive shift and changes in the spectral line shape compared to the pristine catalyst. The spectra are more consistent with the formation of CoOOH and NiOOH species on the surface. Concurrently, the intensity of the Se 3d signal substantially decreased, suggesting partial oxidation or dissolution of surface selenium species. Fig. S5, SEM images reveal that the carbon nanotube framework remains intact, confirming its structural stability. The metal nanoparticles encapsulated within the CNTs are still present. Based on this multi-technique analysis, we conclude that the real active material for OER is the *in situ* formed Co/Ni (oxy)hydroxide layer on the surface, while the selenide core and the NCNT support function as a stable pre-catalyst and conductive backbone, respectively.

**Fig. 5:**
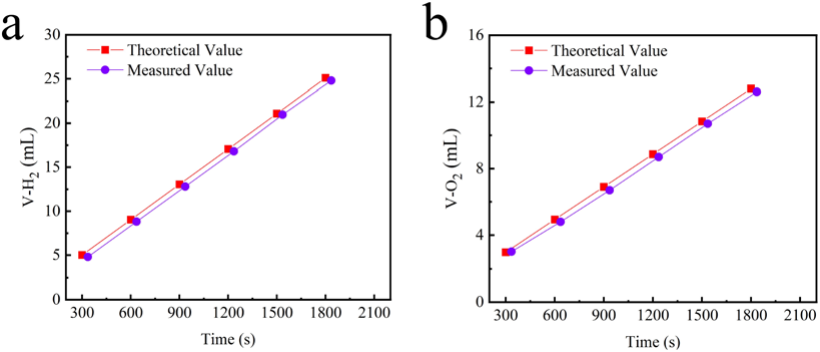
Faradaic efficiency measurements for (a) HER and (b) OER of the Se–CoNi@NCNTs material.

**Fig. 6:**
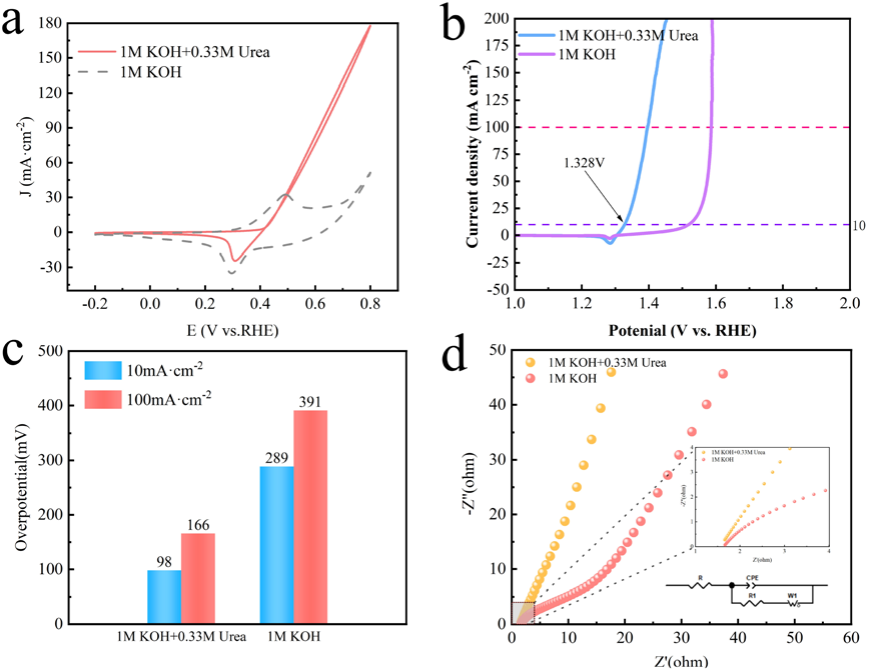
Comparative performance of Se–CoNi@NCNTs material in 1 mol L^−1^ KOH and 1 mol L^−1^ KOH + 0.33 mol L^−1^ urea electrolytes: (a) CV curve, (b) LSV curve, (c) overpotential performance comparison, (d) EIS curve.

As shown in Fig. 5(a and b), the Faraday efficiency of the Se–CoNi@NCNTs(+)‖Se–CoNi@NCNTs(−) electrolyte cell was tested. It is evident that both the anode OER and cathode HER faraday efficiencies approach 100%, sufficiently demonstrating the material's high energy utilisation efficiency. The ratio of actual hydrogen to oxygen produced closely matched the theoretical value of approximately 2 : 1 (Fig. 6), indicating high energy utilisation efficiency. As depicted in Fig. 4(i), the Se–CoNi@NCNTs(+)‖Se–CoNi@NCNTs(−) electrolyte cell achieved oxygen evolution at 10 mA cm^−2^ with a driving voltage of merely 1.655 V (*vs.* RHE), demonstrating exceptional overall water splitting performance.

Fig. 6(a) presents a comparison of the cyclic voltammetry (CV) curves for Se–CoNi@NCNTs in 1 M KOH electrolyte with and without the addition of 0.33 M CO(NH_2_)_2_. The figure demonstrates a marked difference in the oxidation peaks observed in the two electrolytes, confirming that the oxidation reaction occurring after the addition of 0.33 M CO(NH_2_)_2_ involves a complex six-electron transfer process. Fig. 6(b) presents the comparison of LSV curves for Se–CoNi@NCNTs in 1 M KOH electrolyte with and without the addition of 0.33 M CO(NH_2_)_2_. Evidently, the Se–CoNi@NCNTs catalyst achieves UOR performance at merely 1.328 V (*vs.* RHE) to attain a current density of 10 mA cm^−2^ in the 1 M KOH + 0.33 M CO(NH_2_)_2_ electrolyte. In contrast, the OER under the same conditions requires 1.519 V (*vs.* RHE) at the same current density in 1 M KOH electrolyte. This comparison demonstrates markedly faster kinetics for UOR, indicating that substituting UOR for OER represents a viable strategy for significantly reducing catalytic energy consumption. Fig. 6(c) displays the overpotentials at specific current densities (10 mA cm^−2^ and 100 mA cm^−2^) for different electrolytes. Notably, the Se–CoNi@NCNTs catalyst requires only a very low overpotential (166 mV) to achieve a high current density of 100 mA cm^−2^ in the 1 M KOH + 0.33 M CO(NH_2_)_2_ electrolyte. Fig. 6(d) also displays the corresponding Nyquist plot (EIS).

**Fig. 7:**
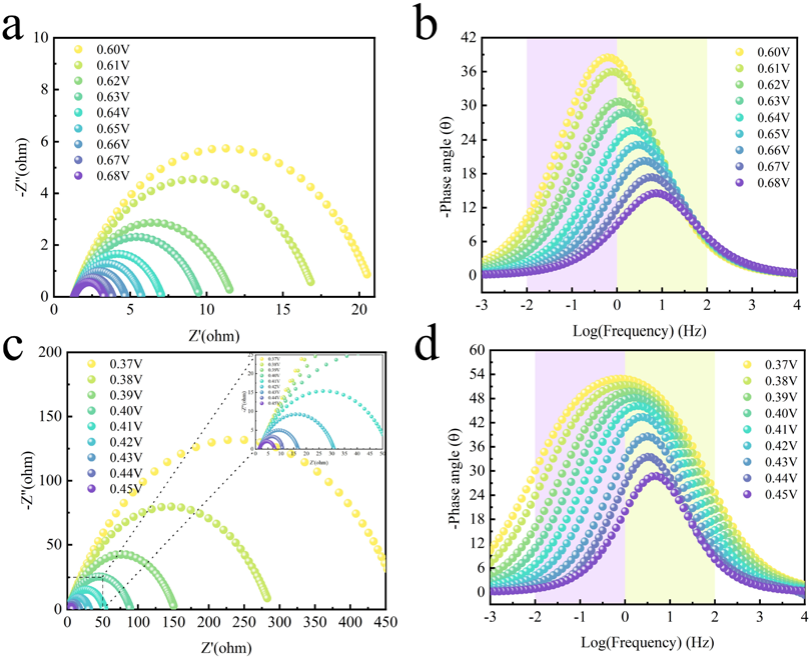
*In situ* electrochemical impedance spectra of Se–CoNi@NCNTs material recorded at different potentials in a 1 mol L^−1^ KOH electrolyte (a) Nyquist plot, (b) Bode plot. *In situ* electrochemical impedance spectra of Se–CoNi@NCNTs material recorded at different potentials: (c) Nyquist plot, (d) Bode plot.

*In situ* impedance spectroscopy serves as an effective method for understanding electrochemical reaction kinetics, particularly concerning the adsorption and desorption kinetics of reactants at electrode surfaces. The high-frequency region (HF) relates to oxidation within the electrode, whilst the low-frequency region (LF) pertains to non-uniform charge distribution caused by oxidised species present at the electrode interface. Consequently, *in situ* electrochemical impedance spectroscopy (EIS-Nyquist, EIS-Bode) tests were conducted at various voltages in electrolytes comprising 1 M KOH with or without the addition of 0.33 M CO(NH_2_)_2_ (Fig. 7). Consistent trends were observed across both solutions, indicating no alteration in the active species. The absence of phase angle peaks in the low- and high-frequency regions of the Bode plots (Fig. 7(b and d)) between 0.6–0.62 V (*vs.* RHE) likely relates to non-uniform charge distribution caused by surface oxidation products. It is discernible that the catalyst's electro-oxidation occurs at the boundary between low and high frequencies, indicating the low-frequency interface resides between the diffusion double layer (DDL) and the catalyst surface. Thus, the OER and UOR of Se–CoNi@NCNTs take place on the electrocatalyst surface. As EIS transitions to the high-frequency interface, NiOOH and CoOOH may form within Se–CoNi@NCNTs, suggesting that the later stages of OER and UOR occur on NiOOH and CoOOH derived from the electro-oxidation of the catalyst surface. The Nyquist plots are shown in Fig. 7(a and c). The Nyquist plot provides insights into the catalyst kinetics and mass transfer characteristics, revealing the relationship between electrical measurements and chemical transformations during the reaction process. *R*_ct_ relates to charge transfer at the electrode interface, while *R*_s_ represents the solution resistance. Consequently, the reaction kinetics of direct reduction are assessed from the relationship between *R*_ct_ and the applied potential. Below the onset potential (0.64 V *vs.* RHE), *R*_ct_ remains elevated, indicating weak charge transfer at the electrode-reaction interface. Above 0.64 V *vs.* RHE, a steep decrease in *R*_ct_ signifies direct oxygen reduction occurring at the electrode surface, consistent with the Bode plot results.

## Conclusions

In summary, we employed a one-step pyrolysis method to synthesise CoNi-composite NCNTs, subsequently utilizing gravity-guided chemical vapour deposition to fabricate a bifunctional electrocatalyst (Se–CoNi@NCNT) on the CoNi@NCNT substrate. This material served as a high-performance cathode and anode electrocatalyst in urea electrolysis cells for hydrogen production. As the anode for UOR, Se–CoNi@NCNT required only a 98 mV overpotential to drive a current density of 10 mA cm^−2^ in alkaline media. This catalyst not only exhibits outstanding OER and UOR performance but also maintains stable material properties for 100 hours at a current density of 50 mA cm^−2^. Furthermore, from a practical application perspective, this catalyst lays the foundation for rationally designing high-performance, low-cost electrocatalysts for urea-containing wastewater electrolysis. These findings present opportunities for the rational design and synthesis of low-cost bifunctional electrocatalysts for sustainable clean energy devices.

## Author contributions

Wenbin Jia: writing – original draft, investigation, data curation, validation. Pengju Wu: investigation, and validation, writing – review & editing. Chao Wu, Meng Yang and Ying Wu: investigation, conceptualization, supervision, project administration, writing – review & editing. All authors discussed the results and provided input to the manuscript.

## Conflicts of interest

The authors declare no competing financial interest.

## Supplementary Material

RA-016-D5RA08405A-s001

## Data Availability

The data supporting this article have been included as part of the supplementary information (SI). Supplementary information is available. See DOI: https://doi.org/10.1039/d5ra08405a.
