# Supplementary material for: Preparation of Se-doped Co/Ni bimetallic composite carbon nanotubes and investigation of their oxidation properties
Source: RSC Adv. 2026 Feb 24;16(12):10761–8. doi: 10.1039/d5ra08405a (PMC12930328; doi:10.1039/d5ra08405a)
Supplement: RA-016-D5RA08405A-s001 [file RA-016-D5RA08405A-s001.pdf]

Supplementary Information (SI) for RSC Advances.
This journal is © The Royal Society of Chemistry 2026

Preparation of Se-Doped Co/Ni Bimetallic Composite Carbon Nanotubes and Investigation of Their Oxidation Properties

Wenbin Jia,*[a] Pengju Wu,[a] Chao Wu,[a] Meng Yang,[a] Ying Wu*[a, b]

[a.]College of Chemistry and Chemical Engineering, Tarim University, Alar, Xinjiang 843300.
[b.]Engineering Laboratory of Chemical Resources Utilization in South Xinjiang, Xinjiang Production & Construction Corps. Alar, Xinjiang 843300.

*Corresponding author. E-mail:
wuyingjuyuan@163.com

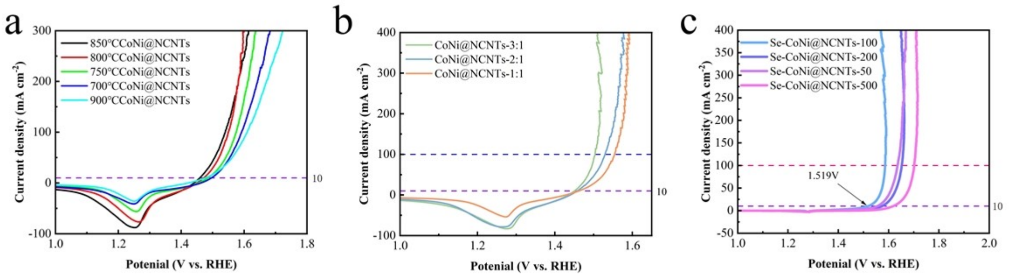

Figure S1 (a) Linear sweep voltammetry (LSV) curves of CoNi@NCNTs composites synthesised at different temperatures; (b) LSV curves of CoNi@NCNTs composites synthesised with varying Co/Ni ratios; (c) LSV curves of Se-CoNi@NCNTs composites with different selenium (Se) additions.

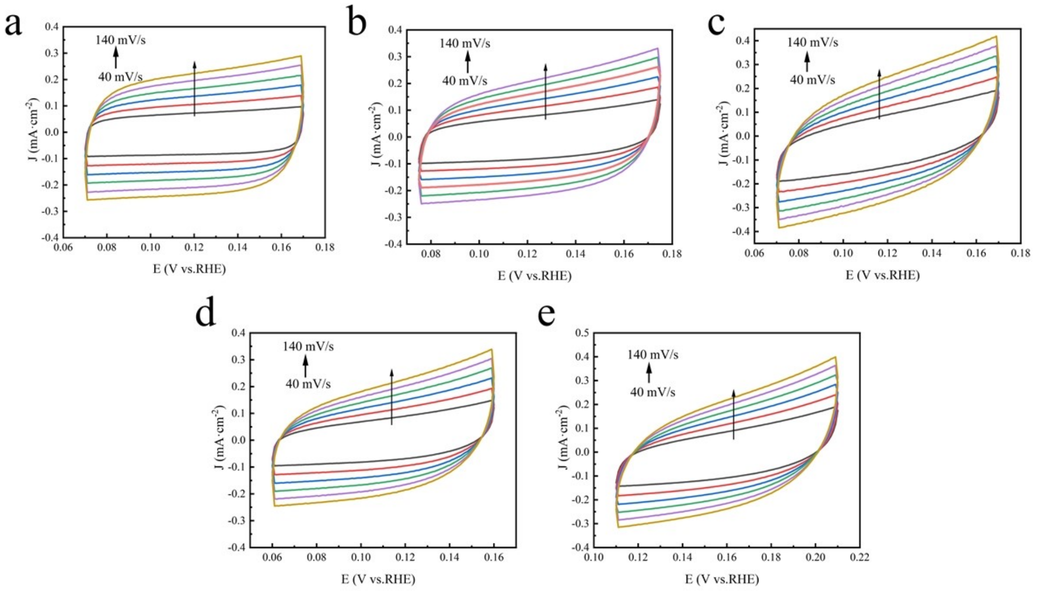

Figure S2 (a) NF, (b) Co@NCNTs, (c) Ni@NCNTs, (d) CoNi@NCNTs, (e) Se-CoNi@NCNTs ECSA-CV curves at different scan rates

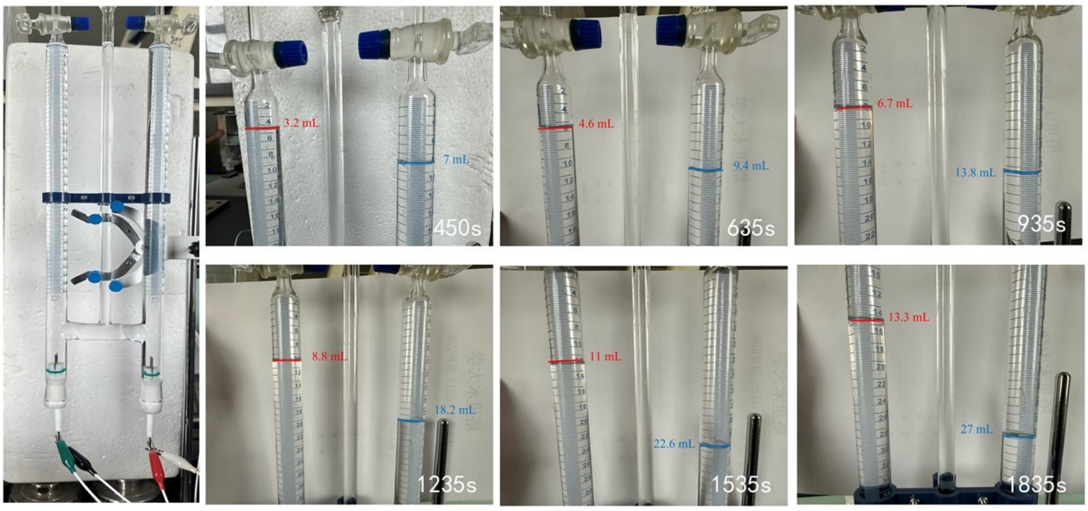

Figure S3: Photograph of the hydrogen and oxygen production testing apparatus for the Hoffmann drainage method

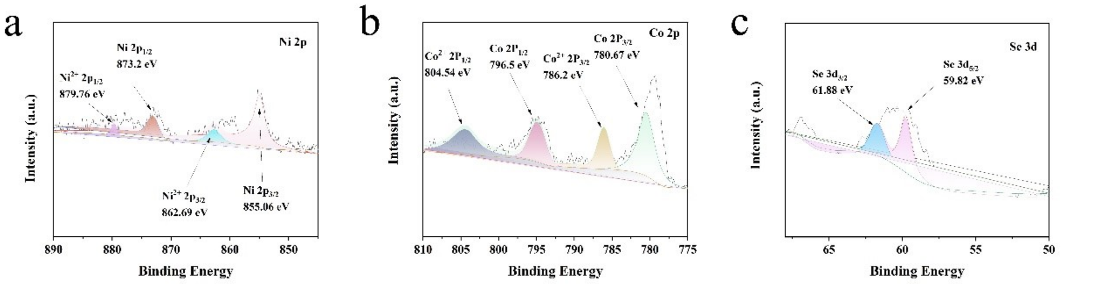

Figure S4: XPS spectra of Se-CoNi@NCNTs after the 100-hour stability test: (a) Ni 2p, (b) Co 2p, (c) Se 3d.

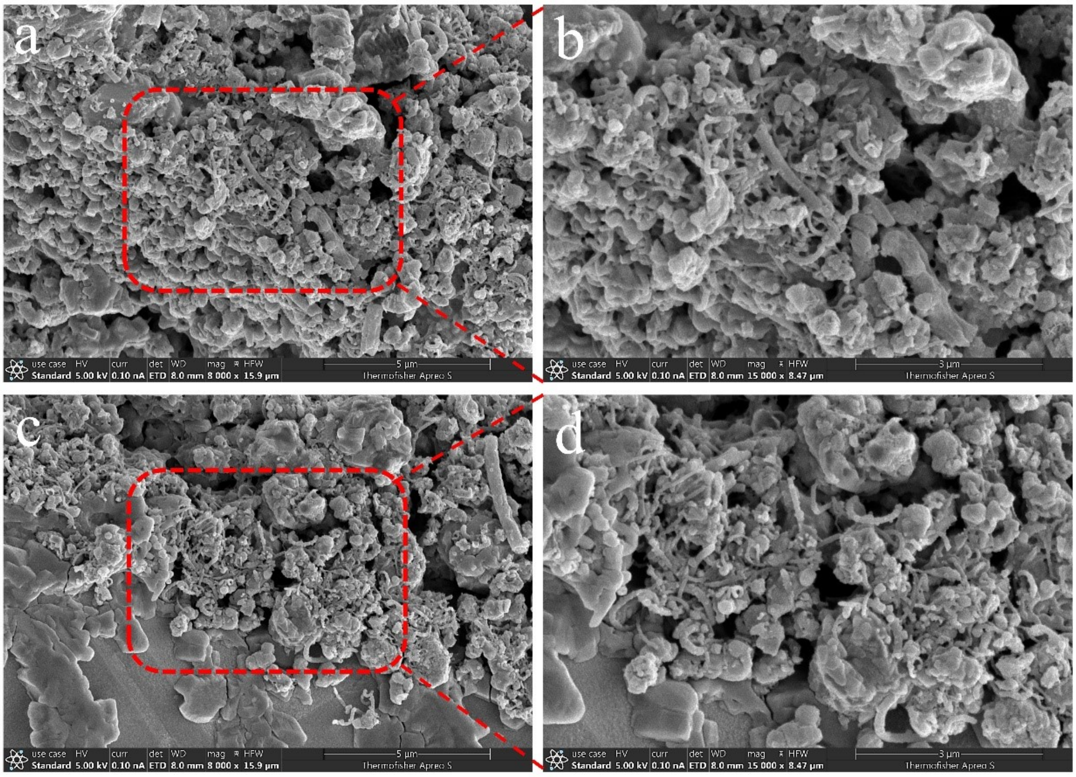

Figure S5: SEM spectra of Se-CoNi@NCNTs after the 100-hour stability test
